# High rates of clinically relevant incidental findings by total-body CT scanning in trauma patients; results of the REACT-2 trial

**DOI:** 10.1007/s00330-016-4598-6

**Published:** 2016-10-05

**Authors:** K. Treskes, S. A. Bos, L. F. M. Beenen, J. C. Sierink, M. J. R. Edwards, B. J. A. Beuker, G. S. R. Muradin, J. Hohmann, J. S. K. Luitse, M. W. Hollmann, M. G. W. Dijkgraaf, J. C. Goslings, on behalf of the REACT-2 study group, T. P. Saltzherr, T. Schepers, V. M. de Jong, R. van Vugt, M. Brink, J. Peters, M. El Moumni, J. S. Harbers, K. W. Wendt, E. M. M. van Lieshout, M. J. Elzinga, E. H. Jansen, C. Zähringer, N. Bless, R. Bingisser

**Affiliations:** 1 Trauma Unit, Department of Surgery, Academic Medical Center, Meibergdreef 9, 1105 AZ Amsterdam, The Netherlands; 2 Department of Radiology, Academic Medical Center, Meibergdreef 9, 1105 AZ Amsterdam, The Netherlands; 3 Department of Trauma and emergency surgery, Radboud University Medical Center, Geert Grooteplein-Zuid 10, 6525 GA Nijmegen, The Netherlands; 4 Trauma Unit, Department of Surgery, University Medical Center Groningen, Hanzeplein 1, 9700 RB Groningen, The Netherlands; 5 Department of Radiology, Erasmus MC, University Medical Center Rotterdam, ‘s-Gravendijkwal 230, 3015 CE Rotterdam, The Netherlands; 6 Department of Radiology and Nuclear Medicine, University of Basel Hospital, Petersgraben 4, 4031 Basel, Switzerland; 7 Department of Anaesthesiology, Academic Medical Center, Meibergdreef 9, 1105 AZ Amsterdam, The Netherlands; 8 Clinical Research Unit, Academic Medical Center, Meibergdreef 9, 1105 AZ Amsterdam, The Netherlands

**Keywords:** Total-body CT, Incidental findings, Multiple trauma, Wounds and injuries, Multidetector computed tomography

## Abstract

**Objectives:**

To determine whether there is a difference in frequency and clinical relevance of incidental findings detected by total-body computed tomography scanning (TBCT) compared to those by the standard work-up (STWU) with selective computed tomography (CT) scanning.

**Methods:**

Trauma patients from five trauma centres were randomized between April 2011 and January 2014 to TBCT imaging or STWU consisting of conventional imaging with selective CT scanning. Incidental findings were divided into three categories: 1) major finding, may cause mortality; 2) moderate finding, may cause morbidity; and 3) minor finding, hardly relevant. Generalized estimating equations were applied to assess differences in incidental findings.

**Results:**

In total, 1083 patients were enrolled, of which 541 patients (49.9 %) were randomized for TBCT and 542 patients (50.1 %) for STWU. Major findings were detected in 23 patients (4.3 %) in the TBCT group compared to 9 patients (1.7 %) in the STWU group (adjusted rate ratio 2.851; 95%CI 1.337–6.077; *p* < 0.007). Findings of moderate relevance were detected in 120 patients (22.2 %) in the TBCT group compared to 86 patients (15.9 %) in the STWU group (adjusted rate ratio 1.421; 95%CI 1.088–1.854; *p* < 0.010).

**Conclusions:**

Compared to selective CT scanning, more patients with clinically relevant incidental findings can be expected by TBCT scanning.

***Key points*:**

• *Total*-*body CT scanning in trauma results in 1.5 times more incidental findings*.

• *Evaluation by TBCT in trauma results in more patients with incidental findings*.

• *In every category of clinical relevance*, *TBCT detects more incidental findings*.

## Introduction

Total-body computed tomography scanning (TBCT) is often used during the primary assessment of patients after severe trauma. Instead of selective computed tomography (CT) scanning of specific body regions, trauma teams routinely perform CT scans of the head, neck, thorax, abdomen and pelvis for trauma patients who could benefit from TBCT scanning. A potential disadvantage compared to the selective approach is the increased radiation exposure of TBCT scanning [1, 2]. Since TBCT has not been shown to reduce mortality in the general trauma population, indication setting is important and a subject of debate [3]. Another consideration when performing TBCT is the increase in non-trauma-related radiologic findings. These concomitant incidental findings should be prioritized with respect to potential life-threatening injuries, and may require additional follow-up and treatment. Incidental findings could provide the advantage of earlier diagnosis of malignancy or vascular disease. On the contrary, when clinical significance is absent, incidental findings could also result in unnecessary investigations and concerns for the patient and extra health care costs.

Previous studies have reported the detection of incidental findings in 32 % to 43 % of trauma patients screened in trauma centres with selective CT scanning [4, 5], and studies on TBCT scanning have reported incidental findings in 45 % to 53 % of trauma patients [6–8]. However, direct comparisons of the frequency and relevance of incidental findings between TBCT and standard work-up with selective CT scanning in trauma patients are lacking. A difference in the frequency of relevant incidental findings can therefore only be assumed.

The aim of this study was to determine whether incidental findings detected by TBCT scanning differed in frequency and relevance from those detected by conventional imaging supplemented with selective CT in patients with severe trauma.

## Patients and methods

### Study design and patient selection

This study was a secondary analysis of patients selected for the REACT-2 multicentre randomized controlled trial, of which the study protocol and main results were published previously [3, 9]. In short, in the REACT-2 trial, adult trauma patients with compromised vital parameters, clinical suspicion of specific severe injuries or high-risk trauma mechanisms were randomized to undergo either an immediate TBCT scan or standard radiological work-up (STWU). The inclusion and exclusion criteria are listed in the table 4 in the appendix. Patients included those seen between April 2011 and January 2014 in four level 1 trauma centres in the Netherlands and one in Switzerland. Informed consent was temporarily waived during the initial presentation in the trauma room. At the earliest opportunity after the trauma, patients or their legal representatives were given work-up information and informed consent was requested. The study was approved by the medical ethics committees at all participating centres (AMC MEC 10/145).

The randomization process was performed by the trauma team immediately after the primary assessment of the patient. TBCT scanning was performed without conventional imaging or sonography in advance and consisted of a non-enhanced CT scan of the head and neck, with arms alongside the trunk, followed by a contrast-enhanced CT scan of the chest, abdomen and pelvis. The preferred technique for the second scan was split-bolus intravenous contrast imaging after raising of the arms, if possible [10]. STWU consisted of x-rays of the chest and pelvis, a focused assessment with sonography for trauma (FAST), and CT scans from specific body regions if indicated. Indications for selective CT scanning were predefined according to local protocols. These indications are listed in the table 4 in the appendix. CT scanners were located in the trauma room or in an adjacent room, and all were 64-slice multidetector row CT scanners.

### Data collection

Radiological images were interpreted by the radiology resident and subsequently by a senior radiologist experienced in trauma imaging. Although focusing on traumatic injuries, this ‘double-reading system’ minimizes the number of missed findings [11]. All findings were described in the radiological reports, which are accessible through the computerized hospital databases of participating centres. Any available previous radiologic imaging of the same patient was also reviewed to confirm the findings to be new. In addition, trauma room reports, interventional and pathology reports, and discharge letters were reviewed. For follow-up data, all available and relevant in-hospital files were searched within a minimum of 6 months and maximum of 2 years after admission to the trauma resuscitation room.

### Definitions and categorization of incidental findings

The clinical relevance of an incidental finding was divided into three subcategories: 1) major finding, may cause mortality; 2) moderate finding, may cause morbidity; and 3) minor finding, hardly relevant and no follow-up needed. The findings and corresponding relevance were scored based on the latest information on the finding. Using earlier reports on this subject, a list of incidental findings that could be expected was formulated before data acquisition [4–8, 12, 13]. Some incidental findings were added to more than one relevance category, as the clinical importance of the same type of finding varied widely. For these specific findings, size-, age- or complexity-specific cut-off values have been added to their description (e.g. simple vs. complicated renal cyst). Findings that had been described previously, as well as traumatic lesions, were excluded. Degenerative joint diseases, common atherosclerotic vessel disease, enostosis, sinusitis, age-related cerebral atrophy, and signs of earlier operations or old cerebral hematoma/infarction were also excluded as incidental findings, in accordance with previous literature [4, 8, 14].

For pulmonary nodules and renal cysts, classification was performed in accordance with the Fleischner society pulmonary nodule recommendations and Bosniak renal cyst classification [15, 16]. With respect to the Fleischner recommendations, findings in patients requiring follow-up within 6 months were defined as major findings, follow-up between 6 and 12 months as moderate findings, and minor if no follow-up was needed. Bosniak class 1 corresponds to minor findings, class 2 to moderate findings, and classes 2 F and over to major findings. In the case of any abnormal lymph nodes, asymptomatic findings were classified as minor unless the nodes were >10 mm in size, in which case they were labelled as moderate. Lymphadenopathy of major relevance indicates suspected lethal findings such as a suspected (non-)Hodgkin lymphoma. If the nodular size was not reported, classification was performed by the reviewers of this study according to follow-up advice or further descriptions from the reporting radiologist.

Multitrauma patients were defined as patients with an Injury Severity Score ≥16. Traumatic Brain Injury (TBI) patients were defined as patients with a Glasgow Coma Scale (GCS) score <9 at presentation and Abbreviated Injury Scale (AIS)-Head code ≥3.

### Statistical analysis

Continuous data with a normal distribution are presented as means with standard deviation, and the non-normally distributed data are presented as medians with interquartile ranges (IQR). Independent samples *t* tests and Mann–Whitney *U* tests were used to compare the parametric and non-parametric continuous data, respectively. Generalized estimating equations (GEE) were applied to assess differences in incidental findings between TBCT and STWU. The Kolmogorov–Smirnov test was used to confirm Poisson-distributed numbers of major and moderate incidental findings. The total number of incidental findings, as well as the number of minor incidental findings, seemed to be negative binomially distributed, which was confirmed by a non-significant chi-square test of goodness of fit of observed and theoretical data, the latter generated with the ‘rnegbin’ function in the R computing environment. The results are reported as rate ratios of incidental findings with TBCT versus STWU, corrected for age, sex and centre. The chi-square test was used to compare categorical variables when categories consisted of at least ten cases; otherwise, Fisher’s exact test was used. A *p* value below 0.05 was considered to reflect statistical significance. Statistical analysis was performed with SPSS version 23 software (IBM Corp., Armonk, NY, USA).

## Results

Baseline characteristics of the study population are shown in Table 1. In total, 1083 patients were enrolled, of which 541 patients were randomized for TBCT (*n* = 49.9 %). The median age was 42 years (IQR 27–59) in the TBCT group and 45 years (IQR 26–59) in the STWU group (*p* = 0.746). The baseline characteristics were comparable between groups except for the number of multitrauma patients: TBCT *n* = 362 (66.9 %) vs. STWU *n* = 331 (61.1 %), *p* = 0.045. Median ISS did not differ between groups: TBCT 20 (IQR 10–29) vs. STWU 19 (9–29), *p* = 0.405.

**Table 1:** Baseline demographic and clinical characteristics of patients*

Characteristic	Total-body CT (*n* = 541)	Standard work-up (*n* = 542)
Age (years)	42 (27–59)	45 (26–59)
Male sex, *n* (%)	413 (76.3)	411 (75.8)
Blunt trauma, *n* (%)	530 (98.0)	534 (98.5)
Trauma mechanism blunt trauma, n (%)		
Fall from height	170 (32.1)	178 (33.3)
MVC – patient as occupant	201 (37.9)	190 (35.6)
MVC – patient as cyclist	65 (12.3)	60 (11.2)
MVC – patient as pedestrian	29 (5.5)	45 (8.4)
Other	65 (12.3)	61 (11.4)
Comorbidity, *n* (%)		
ASA I or II	495 (95.7)	501 (96.2)
ASA III, IV or V	22 (4.3)	20 (3.8)
CT performed at ED, *n* (%)*		
Head	539 (99.6)	483 (89.1)
Neck	535 (98.9)	480 (88.6)
Chest	529 (97.8)	315 (58.1)
Abdomen/pelvis	528 (97.6)	278 (51.3)
Abbreviated Injury Scale ≥3, *n* (%)		
Head	247 (45.7)	218 (40.2)
Chest	229 (42.3)	206 (38.0)
Abdomen/pelvic content	49 (9.1)	67 (12.4)
Pelvis and extremities	150 (27.7)	154 (28.4)
Injury Severity Score (points)	20 (10–29)	19 (9–29)
Multitrauma patients, *n* (%)*^†^	362 (66.9)	331 (61.1)
TBI patients, *n* (%)^†^	178 (32.9)	151 (27.9)

* Results in this table were published earlier [3]. *p* > 0.05 for all between-group comparisons except for CT performed (*p* < 0.001 for all body regions) and multitrauma patients (*p* = 0.045). All data are number (%) or median (interquartile range)

^†^ Multitrauma patients are defined as ISS ≥16. Traumatic Brain Injury (TBI) patients are defined as GCS <9 (Glasgow Coma Scale) at presentation and Abbreviated Injury Scale (AIS)-Head ≥3

*MVC* Motor vehicle collision, *ASA* American Society of Anaesthesiologists, *ED* emergency department

In total, 441 incidental findings were found in 233 of the patients (42.9 %) randomized for TBCT, compared to 290 findings in 167 of the patients (32.5 %) randomized for the STWU (adjusted rate ratio 1.531; 95 % confidence interval [95%CI] 1.274–1.840; *p* < 0.001), as shown in Table 2. Major findings were detected in 23 patients (4.3 %) in the TBCT group, compared to 9 patients (1.7 %) in the STWU group (adjusted rate ratio 2.851; 95%CI 1.337–6.077; *p* < 0.007). Moderate findings were detected in 120 patients (22.2 %) and 86 patients (15.9 %) in the TBCT and STWU groups, respectively (adjusted rate ratio 1.421; 95%CI 1.088–1.854; *p* < 0.010).

**Table 2:** Trauma patients with incidental findings

Characteristic	Total-body CT (*n* = 541)	Standard work-up (*n* = 542)	Rate ratio (95%CI)	*p*	Adjusted rate ratio* (95%CI)	*p*
Patients with incidental findings, n (%)	232 (42.9)	176 (32.5)	1.524 (1.251–1.856)	<0.001^†^	1.531 (1.274–1.840)	<0.001^†^
Major relevance	23 (4.3)	9 (1.7)	2.672 (1.243–5.744)	0.012^‡^	2.851 (1.337–6.077)	0.007^‡^
Moderate relevance	120 (22.2)	86 (15.9)	1.394 (1.051–1.849)	0.021^‡^	1.421 (1.088–1.854)	0.010^‡^
Minor relevance	172 (31.8)	129 (23.8)	1.551 (1.240–1.940)	<0.001^†^	1.536 (1.238–1.905)	<0.001^†^

*Adjusted for age, sex and centre, ^†^ Wald test with assumption of negative binomial distribution, ^‡^ Wald test with assumption of Poisson distribution

95 %CI = 95 % confidence interval

Table 3 shows comparisons of the distribution of the incidental findings over clinical categories between the two groups. Distribution over categories of relevance, body regions, organ systems or neoplasms was similar between the imaging groups. Tables 5 and 6 in the appendix show the follow-up and medical documentation of incidental findings per category of relevance. These characteristics were comparable between the imaging groups; however, follow-up rates were low, and documentation of incidental findings was poor in both groups. In the discharge letters, 39.3 % of the major findings and 13.8 % of the moderate findings were mentioned.

**Table 3:** Characteristics of incidental findings

Characteristic	Total-body CT	Standard work-up	p*
Incidental findings, *n*	441	290	
Body region, n (%)			
Head	56 (12.7)	39 (13.4)	0.786
Neck	26 (5.9)	22 (7.6)	
Thorax	65 (14.7)	47 (16.2)	
Abdomen/pelvis	292 (66.2)	180 (62.1)	
Extremities	2 (0.5)	2 (0.7)	
Organ system, *n* (%)			
Reno-adrenal	112 (25.4)	75 (25.9)	0.696
Hepatobiliary	92 (20.9)	64 (22.1)	
Respiratory	55 (12.5)	27 (9.3)	
Reticuloendothelial	49 (11.1)	33 (11.4)	
Neurological	33 (7.5)	25 (8.6)	
Endocrine	27 (6.1)	21 (7.2)	
Gastrointestinal	27 (6.1)	11 (3.8)	
Urethro-genital	18 (4.1)	15 (5.2)	
Cardiovascular	16 (3.6)	15 (5.2)	
Musculoskeletal	11 (2.5)	4 (1.4)	
Cutaneous	1 (0.2)	0 (0)	
Neoplasm			
Confirmed	20 (4.5)	10 (3.4)	0.456
Suspected	29 (6.6)	14 (4.8)	

* Chi-square test for distribution across categories

The complete list of all findings arranged by body region and relevance is presented in Table 7 in the appendix. Simple renal and hepatic cysts were the most common of all incidental findings for patients in both imaging groups. Suspicious pulmonary nodules were the most described potentially lethal finding (*n* = 6). Of all findings of moderate relevance, gallstones and hepatic steatosis were most frequently described. One in every 24 incidental findings was a pathologically confirmed neoplasm (4.1 %).

## Discussion

This study shows that TBCT imaging is more likely to detect an incidental finding than the standard work-up with selective CT scanning. In every category of clinical relevance, the TBCT scan detected significantly more findings. The incidental findings did not differ in distribution over body regions or tissue types, although the abdominal region showed the largest difference. We could not demonstrate a significant difference in follow-up, which could be explained by low follow-up rates in general and poor documentation of incidental findings and their management in trauma patients. Trauma teams using TBCT scanning should be aware of an increase in relevant incidental findings and should pay special attention to the reporting and management of these additional findings.

In the present study, incidental findings occurred in 43 % of patients undergoing TBCT scanning, of which 42 % could cause serious morbidity. Similar results were reported in previous studies on incidental findings in TBCT scans for initial trauma evaluation. These studies, however, did not directly compare these to incidental findings with selective CT scanning. The study by Hofstetter et al. found incidental findings in 50 % of their patients, with 29 % of these possibly requiring follow-up [7]. In a study by Barett et al., findings were detected in 53 % of all patients by TBCT, 59 % of which required urgent follow-up [6]. Sierink et al. recently reported incidental findings in 45 % of all patients, with possible follow-up required in 31 % [8]. Thus, with the present study included, the percentage of trauma patients with incidental findings detected by TBCT ranges from 43 % to 53 %. Of these findings, 29 % to 59 % have clinical relevance; however, the definition of clinical relevance will be interpreted differently.

Considering future diagnostic work-up, the separate and detailed inclusion of moderate and major findings in the trauma room report's conclusion may help communicate these findings to the general practitioner and other treating physicians. Poor documentation could result in lack of further diagnostic work-up or treatment in other facilities, a structural problem that has been described in previous reports on CT in trauma and emergency imaging [4, 5, 13, 17–20]. Complete and clear documentation thus might save costs in the long term by eliminating repeated work-up for incidental findings. Future research should aim to establish effective methods for proper reporting and management of incidental findings in trauma patients.

### Limitations and strengths

One limitation of the present study is that the categorization of incidental findings into three relevance groups was subject to personal interpretation, as there is no consensus guideline. Discrepancies between previous studies show that specific findings are not always classified in the same category of clinical relevance. To minimize the effect of interpretation, the categorization of expected incidental findings was performed before data acquisition, in accordance with previous literature and under the supervision of an experienced radiologist. The classification system used in this study closely resembled those of previous studies [4, 6–8, 14].

Second, the documentation of incidental findings in the radiology reports could be incomplete. The number of incidental findings may have been influenced by the acute setting of trauma care, and therefore findings of minor or moderate interest may not have been reported at all, since they seemed irrelevant during primary trauma care. However, the risk of underestimation was reduced by the double-reading system. On the other hand, the rate of unknown findings might be overestimated, because previous imaging of the patient might not have been available during formulation of the radiology report.

Third, the follow-up is likely underestimated due to reporting issues as well. Follow-up was between 6 months and 2 years after the first trauma presentation, and only within the in-hospital documentation of the trauma centres where the patient was initially seen. Subsequently, some patients—for example, those with pulmonary nodules—would receive their first follow-up after 1 year or in a different hospital. Furthermore, it is possible that the finding was discussed and that a watchful waiting approach was preferred but not reported in the patient’s file.

This study, which investigated the frequency and clinical relevance of incidental findings in trauma, is the first to directly compare TBCT scanning with conventional imaging supplemented with selective CT. Additional strengths include the international multicentre setting, large comparable patient groups and its randomization setting. Lastly, the list of expected incidental findings aided in adequate prospective categorization.

## Conclusion

When using TBCT instead of selective CT scanning in primary trauma care, a greater number of clinically relevant incidental findings can be expected. Data did not show a significantly higher workload through follow-up; however, documentation of follow-up was suboptimal. When evaluating trauma patients with TBCT scanning, extra alertness towards detection, documentation and follow-up of relevant incidental findings is warranted.

## Appendix 1 Indications for immediate total-body CT in trauma patients used in REACT-2 trial

Trauma patients with one of the following parameters at hospital arrival:

- respiratory rate ≥30/min or ≤10/min
- pulse ≥120/min
- systolic blood pressure ≤100 mmHg
- estimated exterior blood loss ≥500 ml
- Glasgow Coma Score ≤13
- abnormal pupillary reaction

OR

Patients with a clinical suspicion of one of the following diagnoses:

- fractures from at least two long bones
- flail chest, open chest or multiple rib fractures
- severe abdominal injury
- pelvic fracture
- unstable vertebral fractures/spinal cord compression

OR

Patients with one of the following injury mechanisms:

- fall from a height (>3 meters/>10 feet)
- ejection from a vehicle
- death of occupant in same vehicle
- severely injured patient in same vehicle
- wedged or trapped chest/abdomen

## Contraindications

Trauma patients with any of the following characteristics will be excluded:

- known age <18 years
- known pregnancy
- referred from another hospital
- clearly low-energy trauma with blunt injury mechanism
- any patient with a stab wound in one body region
- any patient who is judged to be too unstable to undergo a CT scan and requires (cardiopulmonary) resuscitation or immediate operation because death is imminent

## Appendix 2

**Table 4:** Indications for selective CT scanning after conventional imaging

CT-brain A patient with trauma of the head and with at least: → 1 major criterion: - EMV ≤13 - loss of consciousness >30 min - haemodynamically unstable - age ≥60 years - high-risk trauma - vomiting - posttraumatic seizure - coagulopathy risk factors (primary or by medication) - focal neurological deficit - >1 point decline in EMV after 1 h - posttraumatic amnesia >4 h - clinical suspicion for skull base or facial fractures → and/or at least 2 minor criteria: - age between 40-60 years - posttraumatic loss of consciousness - posttraumatic amnesia 2-4 h - externally facial injuries without signs of fractures - 1-point decline in EMV after 1 h CT of the cervical spine 1. Always when CT-brain is performed 2. In all patients unless they meet all the Nexus criteria: - no posterior midline cervical spine tenderness - no focal neurological deficit - a normal level of alertness - no evidence of intoxication - no painful distracting injuries X-cervical spine Never indicated. If Nexus deviant: cervical-CT. Chest CT (with iv contrast) 1. Chest gunshot wound with suspicion of transmediastinal route 2. Acute aortic injury 3. Abnormal mediastinum seen at chest radiography. - mediastinal widening - pleural cap (‘apical cap’) - aorta arc unclear enclosed - left main bronchus removed downwards - deviated trachea or oesophagus - filled aortopulmonary window - widened paraspinal line - widened paratracheal line right 3. Relative indications: - type and severity of trauma - fractures of costa 1 or 2 - thoracic spine fracture - posterior sternoclavicular luxation - hesitation about the existence of pneumothorax/pneumomediastinum or pneumopericardium - fractures of the clavicle and shoulder	Abdominal CT (with iv contrast) 1. Penetrating injuries in abdomen, chest and/or flank 2. Deficits found with FAST - intra-abdominal free fluid - suspicion organ injury - suspicion retroperitoneal injury 3. Dislocated pelvic ring fracture and/or dislocated acetabulum fracture 4. Clinical suspicion of intraabdominal injury at physical examination 5. Subjective judgment of severity of injury by trauma leader - combined thoracic and pelvic injury - ‘seatbelt sign’ - chance fracture X-thoracic and lumbar spine Not indicated when chest or abdominal CT is performed (reconstructions can be made) 1. Complaints of the thoracic and lumbar spine 2. Tenderness of the thoracic and lumbar spine in the midline 3. Loss of consciousness 4. Deficits in peripheral neurologic examination 5. Painful distracting injuries Pelvic CT (with iv contrast) 1. All pelvic ring and acetabulum fractures unless conventional imaging is sufficient for adequate diagnosis and treatment 2. After reposition of hip luxation with suspicion of femoral head fractures and/or acetabulum fracture. When CT-abdomen is performed, CT-pelvis is not necessary. Retrograde urethrogram 1. Male patient with severe pelvic injury (type B and C) 2. Bleeding from the meatus, perineal injury or injury of the outer genital organs 3. Penetrating abdominal injury 4. In women only selectively after inspection Imaging of the extremities When fractures/dislocations are suspected: conventional imaging and selective CT

## Appendix 3

**Table 5:** Follow-up for incidental findings

Characteristic	Total-body CT	Standard work-up	*p**
Incidental finding of *major* relevance, *n*	24	9	0.129^‡^
Follow-up, *n* (%)	12 (50.0)	5 (55.6)	
No follow-up, *n* (%)	9 (37.5)	0 (0.0)	
Deceased in-hospital, *n* (%)*	3 (12.5)	4 (44.4)	
Incidental finding of *moderate* relevance, *n*	160	115	0.141^†^
Follow-up, *n* (%)	10 (6.3)	13 (11.3)	
No follow-up, *n* (%)	126 (78.8)	86 (74.8)	
Deceased in-hospital, *n* (%)*	24 (15.0)	16 (13.9)	
Incidental finding of *minor* relevance, *n*	257	166	0.735^†^
Follow-up, *n* (%)	1 (0.4)	1 (0.6)	
No follow-up, *n* (%)	227 (88.3)	141 (84.9)	
Deceased in-hospital, *n* (%)*	29 (11.3)	24 (14.5)	

* Incidental findings in patients who died in-hospital were excluded from this analysis

^†^ Chi-square, ^‡^ Fisher’s exact

## Appendix 4

**Table 6:** Documentation of incidental findings

Characteristic	Total-body CT	Standard work-up	*p*
Incidental finding of *major* relevance, *n*	24	9	
Trauma letter, *n* (%)	10 (41.7)	5 (55.6)	0.697^†^
Discharge letter, n (%)	10 (41.7)	3 (33.3)	0.999^†^
Incidental finding of *moderate* relevance, *n*	160	115	
Trauma letter, *n* (%)	31 (19.4)	21 (18.3)	0.816*
Discharge letter, *n* (%)	20 (12.5)	18 (15.7)	0.455*
Incidental finding of *minor* relevance, *n*	257	166	
Trauma letter, *n* (%)	11 (4.3)	9 (5.4)	0.642^†^
Discharge letter, *n* (%)	13 (5.1)	6 (3.6)	0.632^†^

* Chi-square, ^†^ Fisher’s exact

## Appendix 5

**Table 7:** List of incidental findings in 1083 trauma patients, categorized by body region and clinical relevance

Location			Frequency	Percentage of total findings
Head	Major			
		mass, brain	4	0.5 %
	Moderate			
		aneurysm, brain, <5.5 cm	1	0.1 %
		cranial osteoma	5	0.7 %
		leukoaraiosis <50 years	2	0.3 %
	Minor			
		retention cyst	27	3.7 %
		leukoaraiosis >50 years	25	3.4 %
		brain calcification	16	2.2 %
		arachnoid cyst	10	1.4 %
		brain cyst	2	0.3 %
		cisterna magna, large	1	0.1 %
		colloid cyst	1	0.1 %
		parotid stone	1	0.1 %
Neck	Major			
		-		
	Moderate			
		thyroid nodule	22	3.0 %
		cervical lymphadenopathy	3	0.4 %
		thyroid lesion	6	0.8 %
		goitre/struma	5	0.7 %
		mass, thyroid	1	0.1 %
		oesophageal hyperplasia	1	0.1 %
		thyroid cyst, complicated	1	0.1 %
	Minor			
		cervical lymphadenopathy	5	0.7 %
		thyroid calcification	2	0.3 %
		thyroid cyst, simple	1	0.1 %
Thorax	Major			
		pulmonary nodule, tumorous aspect	6	0.8 %
		mass, breast	1	0.1 %
		penetrating aortic ulcer	1	0.1 %
		pulmonary lesion, tumorous aspect	1	0.1 %
		thoracic lymphadenopathy	1	0.1 %
	Moderate			
		cardiomegaly	10	1.4 %
		pulmonary nodule, relevant	6	0.8 %
		COPD	3	0.4 %
		aneurysm, thoracic, <5,5 cm	1	0.1 %
		atelectasis	2	0.3 %
		gynecomastia	1	0.1 %
		heart valve calcification	1	0.1 %
		intrathoracic struma	1	0.1 %
		mammary nodule	1	0.1 %
		pleural fluid	1	0.1 %
		pleural plaques	3	0.4 %
		pleural thickening	1	0.1 %
		pulmonary consolidation	4	0.5 %
		pulmonary lesion, relevant	2	0.3 %
		sternal hemangioma	1	0.1 %
		thoracic lymphadenopathy	5	0.7 %
	Minor			
		thymus remainder	20	2.7 %
		pulmonary nodule, small nonspecific	13	1.8 %
		azygos lobe	1	0.1 %
		congenital vascular anomalies	3	0.4 %
		pericardial cyst	2	0.3 %
		pericardial effusion	1	0.1 %
		pulmonary cyst, simple	7	1.0 %
		pulmonary granuloma	6	0.8 %
		sebaceous cyst	1	0.1 %
		thoracic lymphadenopathy	8	1.1 %
Abdomen/Pelvis	Major			
		aneurysm, abdominal, >5.5 cm	1	0.1 %
		dissection, abdominal	1	0.1 %
		mass, adrenal	6	0.8 %
		mass, bladder	1	0.1 %
		mass, hepatic	1	0.1 %
		mass, pararectal	1	0.1 %
		mass, para-splenic	1	0.1 %
		mass, renal	4	0.5 %
		pancreatic lesion, complicated	1	0.1 %
		penetrating aortic ulcer	1	0.1 %
	Moderate			
		gallstone	27	3.7 %
		hepatic steatosis	25	3.4 %
		hepatic nodule, relevant	12	1.6 %
		renal cyst, complicated	12	1.6 %
		renal stone	12	1.6 %
		abdominal aortic aneurysm, <5.5 cm	1	0.1 %
		abdominal aortic stenosis	1	0.1 %
		abdominal lymphadenopathy	5	0.7 %
		adrenal hyperplasia	5	0.7 %
		adrenal hypertrophy	1	0.1 %
		adrenal lesion, relevant	6	0.8 %
		adrenal nodule, relevant	5	0.7 %
		aneurysm, abdominal, <5.5 cm	4	0.5 %
		cryptorchidism	3	0.4 %
		cutaneous nodule, relevant	1	0.1 %
		diaphragmatic hernia	3	0.4 %
		hepatic cyst, complicated	4	0.5 %
		hepatic lesion, relevant	6	0.8 %
		hernia, inguinal	1	0.1 %
		hernia, umbilical	1	0.1 %
		horseshoe kidney	3	0.4 %
		hydrocele testis	1	0.1 %
		hydronephrosis	4	0.5 %
		mono-kidney	1	0.1 %
		pancreatic atrophy	7	1.0 %
		pancreatic calcification	3	0.4 %
		pancreatic cyst	3	0.4 %
		pancreatic steatosis	2	0.3 %
		pneumaturia	1	0.1 %
		porcelain gallbladder	1	0.1 %
		prostatic hypertrophy	8	1.1 %
		renal atrophy	2	0.3 %
		renal lesion, complicated	1	0.1 %
		Riedel's hepatic lobe	1	0.1 %
		splenic lesion, relevant	2	0.3 %
		suspected fibromuscular dysplasia	1	0.1 %
		vesical calculus	1	0.1 %
	Minor			
		renal cyst, simple	97	13.3 %
		hepatic cyst, simple	50	6.8 %
		diverticulosis	35	4.8 %
		spleen, accessory	20	2.7 %
		hepatic lesion, simple	11	1.5 %
		abdominal lymphadenopathy	6	0.8 %
		adrenal cyst, simple	2	0.3 %
		adrenal lesion, simple	4	0.5 %
		adrenal nodule, simple	3	0.4 %
		bladder diverticulum	3	0.4 %
		corpus luteum cyst	1	0.1 %
		duplex collecting system	1	0.1 %
		fluid in rectouterine pouch	3	0.4 %
		intestinal malrotation	1	0.1 %
		ovarian cyst, <5 cm	3	0.4 %
		prostatic calcification	10	1.4 %
		renal calcification	1	0.1 %
		renal cortex, thinning	2	0.3 %
		renal ectopia	1	0.1 %
		renal lesion, simple	3	0.4 %
		splenic cyst, simple	4	0.5 %
		urachal cyst	1	0.1 %
		uterine calcification	4	0.5 %
		uterine fibroid	1	0.1 %
Extremities	Major			
		-		
	Moderate			
		bone lytic lesion	2	0.3 %
	Minor			
		bone cyst, simple	1	0.1 %
		bone lesion, simple	1	0.1 %

## Abbreviations

CT: Computed tomography
TBCT: Total-body computed tomography
STWU: Standard work-up
FAST: Focused assessment with sonography for trauma
GEE: Generalized estimating equation
